# Supplementary material for: Ventralis intermedius nucleus anatomical variability assessment by MRI structural connectivity
Source: Neuroimage. 2021 Sep;238:118231. doi: 10.1016/j.neuroimage.2021.118231 (PMC8960999; doi:10.1016/j.neuroimage.2021.118231)
Supplement: Supplementary file 1 [file mmc1.docx]

**
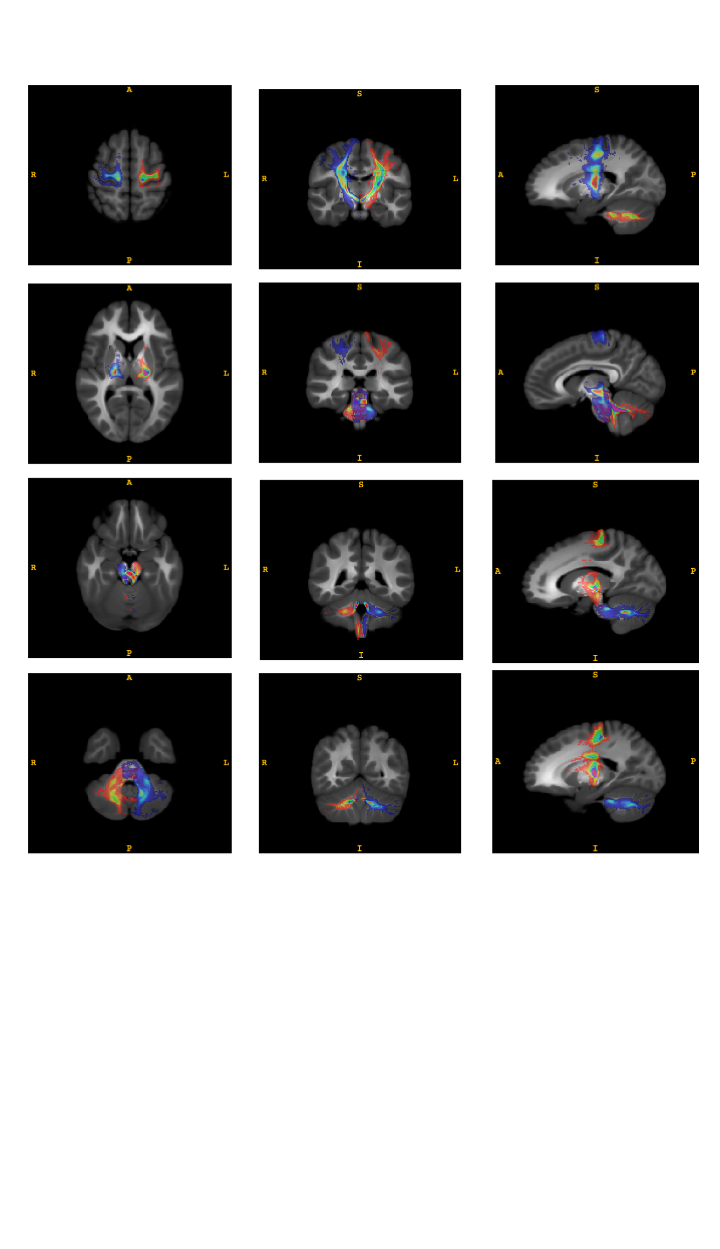
Supplementary material**

**Figure 1.** Percent of subjects with tractography result that survives the threshold, 20% of maximum PICo within the tract-thalamus overlap region.


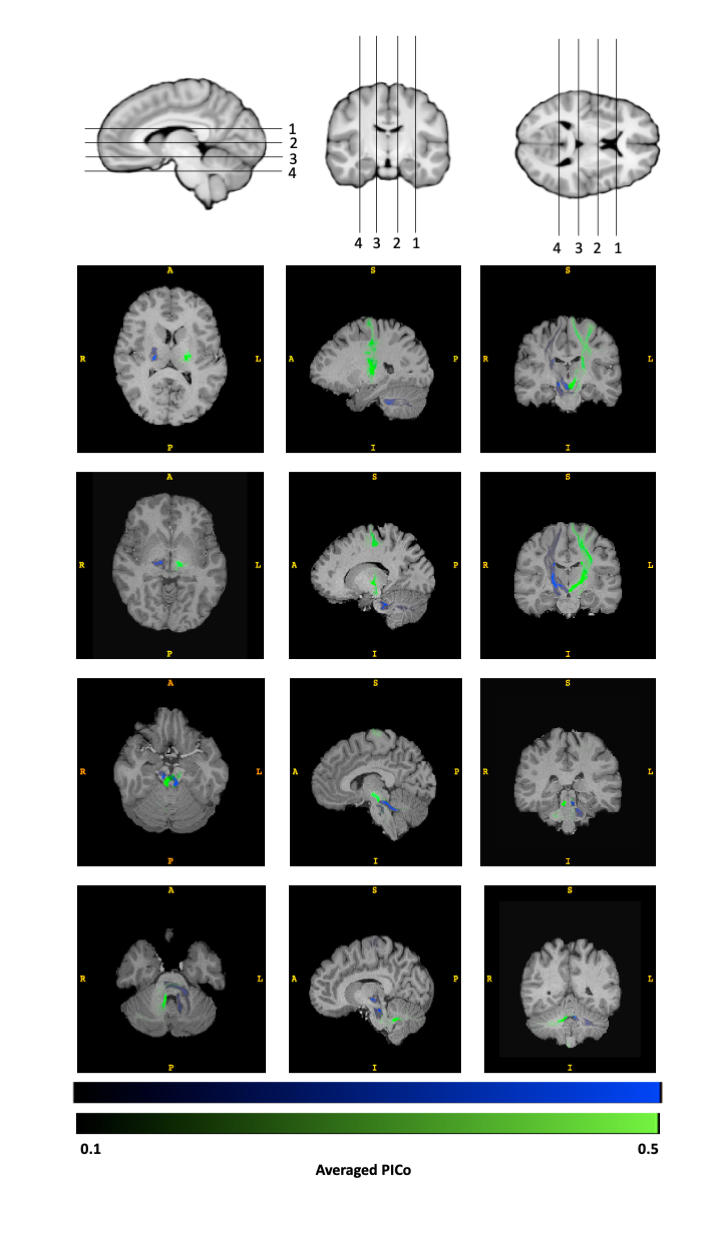


**Figure 2.** Right (blue) and left (green) DTCp for subject 100307, windowed between PICo 0.1 – 0.5


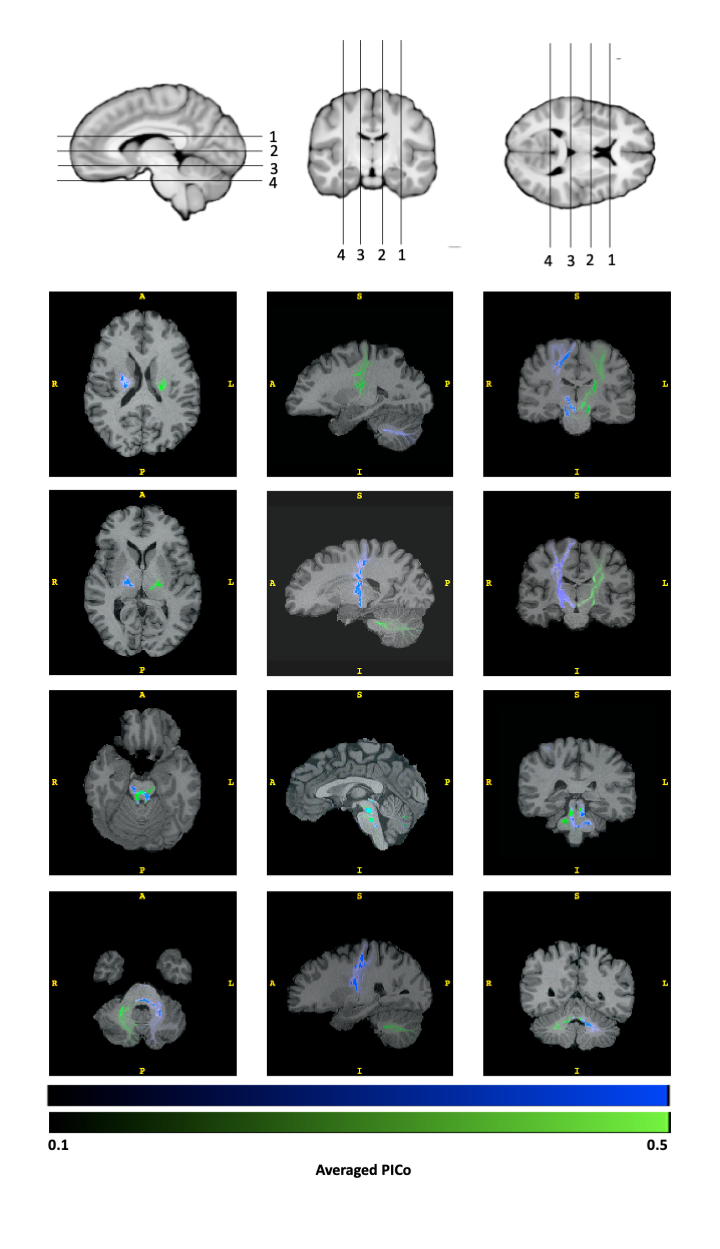


**Figure 3.** Right (blue) and left (green) DTCp for subject 100408, windowed between PICo 0.1 – 0.5


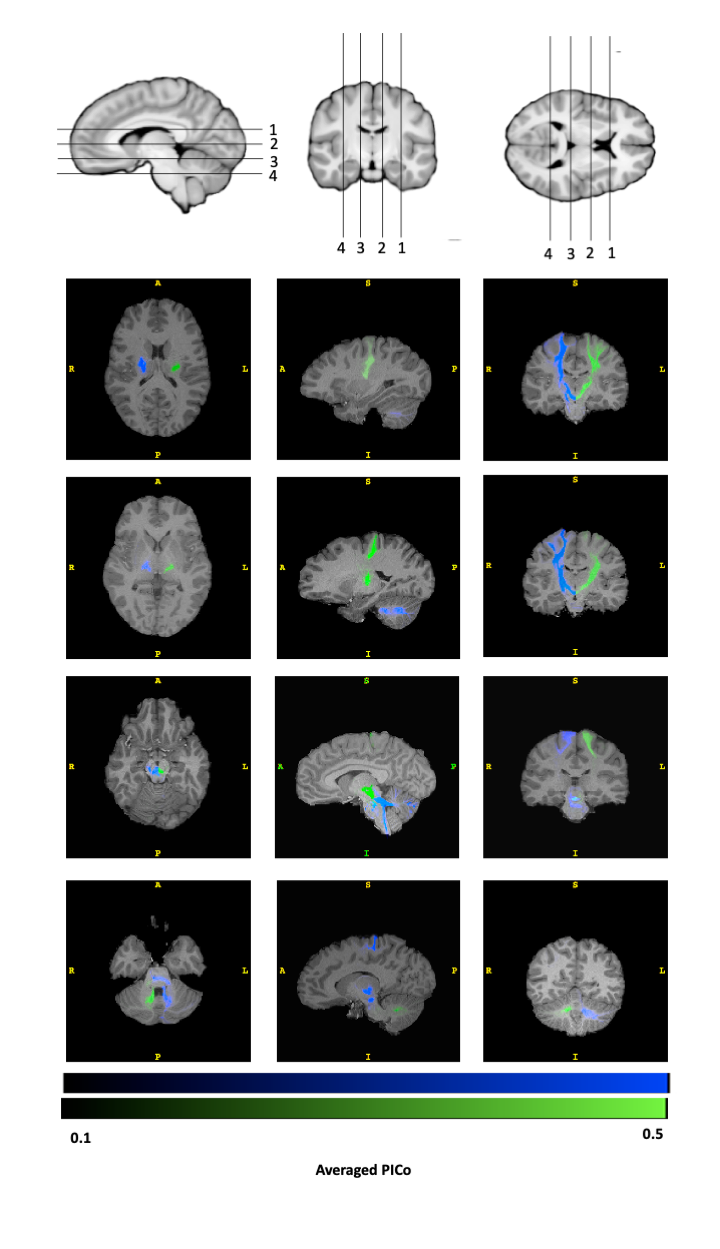


**Figure 4.** Right (blue) and left (green) DTCp for subject 125525, windowed between PICo 0.1 – 0.5


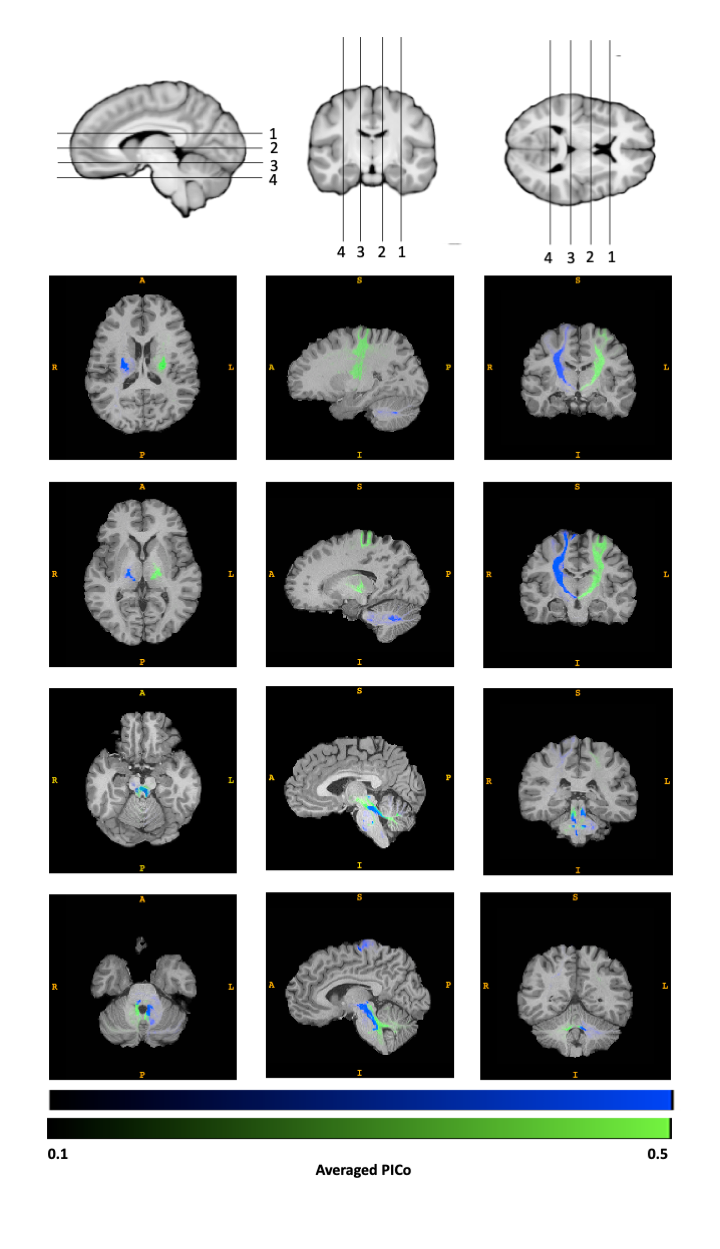


**Figure 5.** Right (blue) and left (green) DTCp for subject 159340, windowed between PICo 0.1 – 0.5


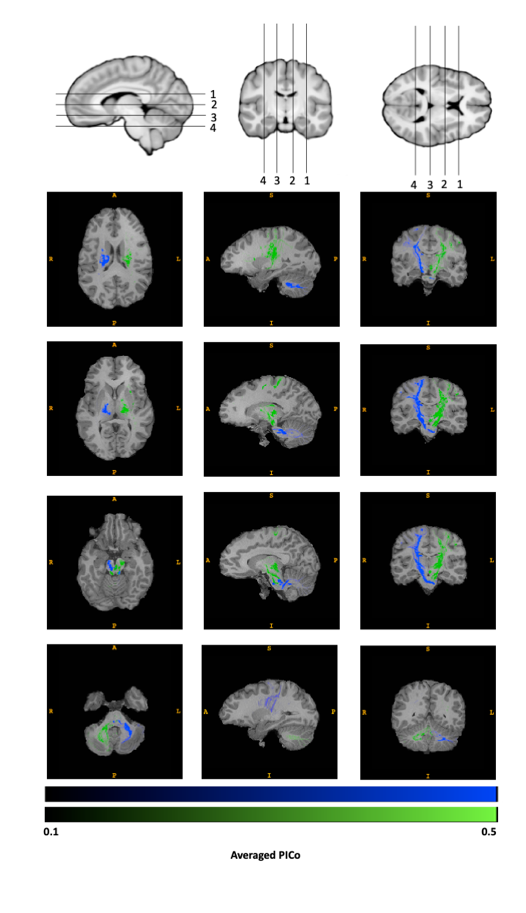


**Figure 6.** Right (blue) and left (green) DTCp for subject 756055, windowed between PICo 0.1 – 0.5
